# Validation and optimization of the Ion Torrent S5 XL sequencer and Oncomine workflow for *BRCA1* and *BRCA2* genetic testing

**DOI:** 10.18632/oncotarget.16799

**Published:** 2017-04-03

**Authors:** Saeam Shin, Yoonjung Kim, Seoung Chul Oh, Nae Yu, Seung-Tae Lee, Jong Rak Choi, Kyung-A Lee

**Affiliations:** ^1^ Department of Laboratory Medicine, Yonsei University College of Medicine, Seoul, Republic of Korea; ^2^ Department of Laboratory Medicine, Hallym University College of Medicine, Kangnam Sacred Heart Hospital, Seoul, Republic of Korea

**Keywords:** BRCA, Ion Torrent, next-generation sequencing, S5 XL, Oncomine

## Abstract

In this study, we validated the analytical performance of *BRCA1/2* sequencing using Ion Torrent's new bench-top sequencer with amplicon panel with optimized bioinformatics pipelines. Using 43 samples that were previously validated by Illumina's MiSeq platform and/or by Sanger sequencing/multiplex ligation-dependent probe amplification, we amplified the target with the Oncomine^™^ BRCA Research Assay and sequenced on Ion Torrent S5 XL (Thermo Fisher Scientific, Waltham, MA, USA). We compared two bioinformatics pipelines for optimal processing of S5 XL sequence data: the Torrent Suite with a plug-in Torrent Variant Caller (Thermo Fisher Scientific), and commercial NextGENe software (Softgenetics, State College, PA, USA). All expected 681 single nucleotide variants, 15 small indels, and three copy number variants were correctly called, except one common variant adjacent to a rare variant on the primer-binding site. The sensitivity, specificity, false positive rate, and accuracy for detection of single nucleotide variant and small indels of S5 XL sequencing were 99.85%, 100%, 0%, and 99.99% for the Torrent Variant Caller and 99.85%, 99.99%, 0.14%, and 99.99% for NextGENe, respectively. The reproducibility of variant calling was 100%, and the precision of variant frequency also showed good performance with coefficients of variation between 0.32 and 5.29%. We obtained highly accurate data through uniform and sufficient coverage depth over all target regions and through optimization of the bioinformatics pipeline. We confirmed that our platform is accurate and practical for diagnostic *BRCA1/2* testing in a clinical laboratory.

## INTRODUCTION

The development of massive-parallel sequencing technology has facilitated the rapid and cost-effective generation of sequence data. Owing to the large size of the target region of *BRCA1/2* genes and no-mutation hot spots, many clinical laboratories are shifting from conventional routine techniques to high-throughput next-generation sequencing (NGS) for *BRCA1/2* testing [1, 2]. However, diagnostic genetic testing in the clinical laboratory requires high accuracy and an acceptable turn-around time for clinical decisions. Therefore, each clinical laboratory should put in place an appropriate NGS process including the wet procedure and bioinformatics analysis that meets the quality standards of clinical genetic testing [3–5].

Bench-top NGS sequencers optimized for targeted sequencing have usually been evaluated for diagnostic *BRCA1/2* testing [1, 6–9]. The latest Ion Torrent sequencers, models S5 and S5 XL (Thermo Fisher Scientific, Waltham, MA, USA), were released in 2015. S5 and S5 XL require much less time for sequencing than previous Ion Torrent Personal Genome Machine (PGM) sequencer (Run time yielding 0.6–1 Gb; 2.5 hr for S5 and S5 XL vs. 4.4 hr for PGM) [10, 11]. Therefore, we can also expect an improvement in turn-around time required for clinical genetic testing.

Several attempts have been made to compare the Illumina and Ion Torrent platforms, which adopt two different principles for library preparation and sequence generation [6, 12, 13]. The Ion Torrent platform, which uses semiconductor sequencing technology, has a reputation for higher insertion/deletion (indel) error rates associated with the homopolymer region than the Illumina platform, which sequences by synthesis technology [12]. However, a number of approaches including optimization of the bioinformatics pipeline, and enhancement of coverage depth and uniformity, have been attempted to overcome the shortcomings of the Ion Torrent platform [1, 9].

The aim of the current study was to validate and optimize the Ion Torrent S5 XL platform using the Oncomine™ BRCA Research Assay (Thermo Fisher Scientific) for routine diagnostic *BRCA1/2* testing in a clinical laboratory. Furthermore, we used previously validated samples from hereditary breast and ovarian cancer syndrome (HBOC) patients for comparison of its performance with the other most commonly used platform: MiSeq sequencer with the TruSeq custom amplicon panel (both Illumina, San Diego, CA, USA) [14]. We also compared two variant calling methods (Torrent Variant Caller v5.2.0.34 *vs* NextGENe v2.4.1.2) for optimal processing of data from the Ion Torrent S5 XL.

## RESULTS

### S5 XL sequencing run metrics

**Figure 1:**
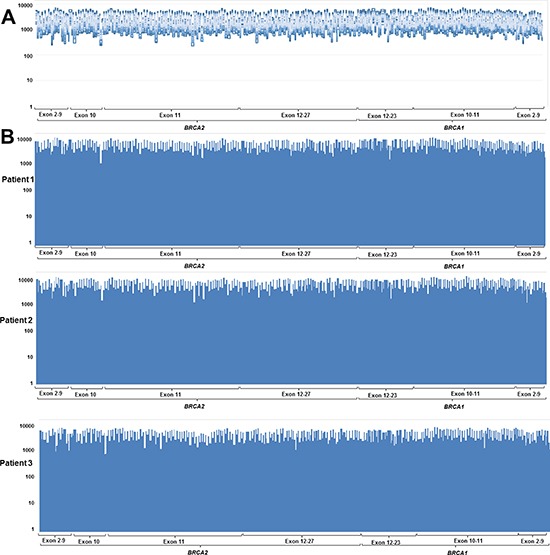
Coverage plots from (**A**) 40 patients without copy number variant and (**B**) three representative samples showed uniform coverage across the entire region of interest. Y-axis indicates sequence read depth and x-axis indicates target

The sequencing run metrics showed acceptable quality in all four batches (Table 1). The percentage of on-target reads, in which the ratio of the number of reads mapped on a target *BRCA1/2* region to the total number of reads, was over 95%. The uniformity of base coverage was over 97% in all batches, and base coverage was over 20 × at all target regions. The sequencing reads covered the region of interest (ROI) evenly (Figure 1).

**Table 1:** S5 XL sequencing run statistics

	Batch 1	Batch 2	Batch 3	Batch 4
On-target reads, %	95.85	95.48	95.65	95.29
On-target base reads, %	93.79	93.27	93.47	92.9
Uniformity of base coverage at 0.2, %	97.69	99.97	99.98	99.63
Average depth per sample (min, max)	1957 × (1062 ×, 3625 ×)	2056 × (1272 ×, 2780×)	2151 × (1639 ×, 2576×)	1944× (1683×, 2114×)
Average depth of on-target regions (min, max)	1834 × (997 ×, 3389 ×)	1834 × (997 ×, 3389 ×)	2010 × (1531 ×, 2411 ×)	1806× (1573×, 1982×)
Target bases with no strand bias, %	98.08	98.76	98.41	98.53
Target base coverage at 20×, %	100	100	100	100

### Accuracy performance of S5 XL sequencing

**Figure 2:**
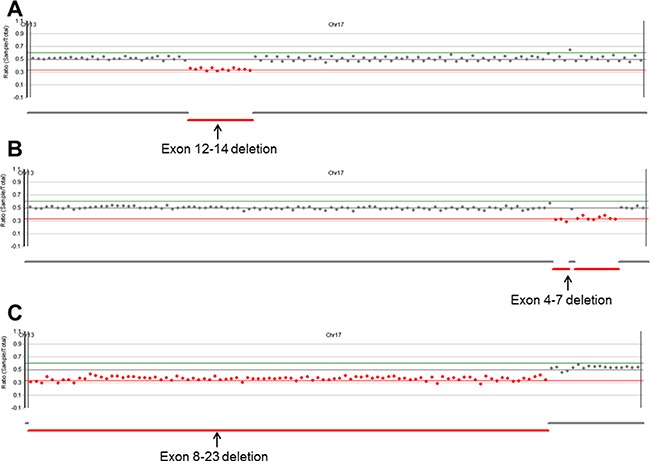
Copy number analysis plots from NextGENe software Our platform successfully detected three CNVs. (**A**) Exon 12-14 deletion, (**B**) exon 4-7 deletion, and (**C**) exon 8-23 deletion in *BRCA1*.

All single nucleotide variants (SNV), small indels, and copy number variants (CNV) were successfully identified, except one SNV with the same false-negative result from the MiSeq platform (variant profile of the 43 samples used for NGS validation are provided in the online version of Supplementary Table 1) [14]. The analytical performance of S5 XL sequencing for SNV and indel calling is summarized in Table 2. Using NextGENe analysis, we discovered one false-positive call (*BRCA2* NM_000059.3: c.8953+15_8953+16insT) in a patient. The error was confirmed as a strand bias with low balance ratio of 0.169. The sensitivity, specificity, false positive rate, and accuracy for SNV and indel calling were 99.85%, 100%, 0%, and 99.99% for Torrent Variant Caller and 99.85%, 99.99%, 0.14%, and 99.99% for NextGENe, respectively. Our NGS platform also detected CNVs from three patients (Figure 2). According to NextGENe analysis, the deleted exon boundaries of each patient were all in agreement with MLPA results.

**Table 2:** Analytical performance of next-generation sequencing compared with Sanger sequencing

Amplicon panel/ Sequencer/variant caller	False positives, *n*	False negatives, *n*	True positives, *n*	True negatives, *n*	Sensitivity, % (95% CI)	Specificity, % (95% CI)	False positive rate, % (95% CI)	Accuracy, % (95% CI)
Oncomine/S5 XL/ Torrent variant caller	0	1	695	920787	99.85 (99.57–100.13)	100	0	99.99 (99.98–99.99)
Oncomine/S5 XL/ NextGENe	1	1	695	920786	99.85 (99.57–100.13)	99.99 (99.89–99.99)	0.14 (−0.13–0.41)	99.99 (99.98–99.99)
TruSeq/MiSeq/GATK	56	1	695	920731	99.85 (99.56–100.14)	99.99 (99.89–99.99)	7.46 (5.58–9.33)	99.99 (99.98–99.99)

GATK = genome analysis toolkit; CI = confidence interval.

**Figure 3:**
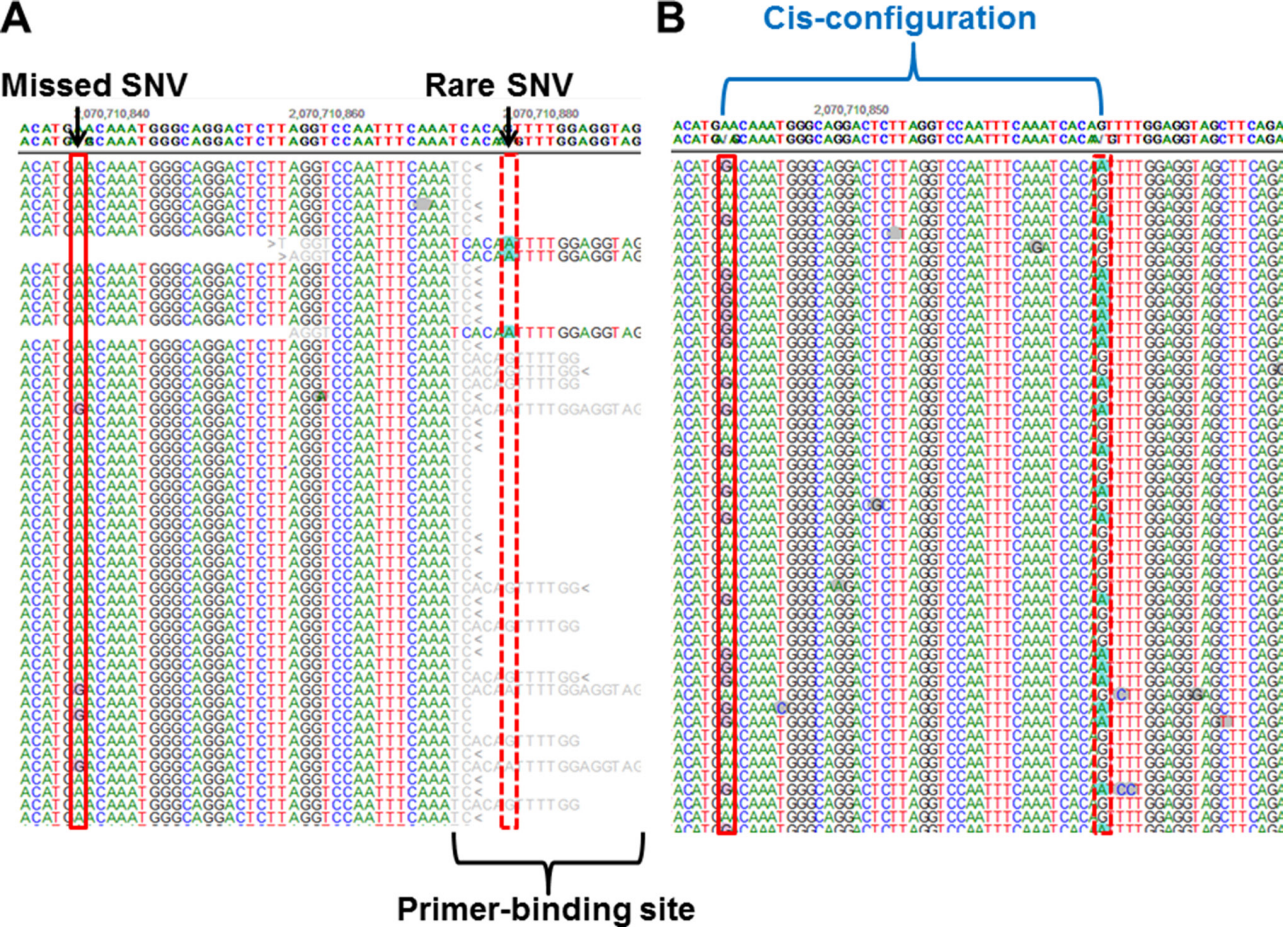
A false-negative variant from a previous study [14] using the MiSeq platform and S5 XL sequencing (**A**) A common single nucleotide variant (SNV) in BRCA2 (c.2971A>G, p.Asn991Asp; red solid bordered box) showed low variant frequency (12.7%) according to S5 XL sequencing owing to a rare SNV (c.3011G>A, p.Ser1044Asn; red dotted bordered box) on the primer-binding site. (**B**) Deep-sequencing using the other probe set revealed that the missed SNV and the adjacent rare SNV were in a cis-configuration.

One missing SNV (c.2971A>G, p.Asn991Asp in a patient, dbSNP ID; rs1799944) was not identified by Oncomine panel on S5 XL or by TruSeq custom panel on MiSeq [14]. After lowering the variant frequency cut-off to ≥ 10%, bioinformatics analysis revealed that the missed SNV was called with a variant frequency of 12.7% by Torrent Variant Caller and 12.4% by NextGENe. In the same batch, the rs1799944 variant was correctly called with 46.9–50.2% variant frequency in four other patients. The missed SNV was covered by two primer sets from the Oncomine panel and one primer was located in the rare individual SNV of one patient (c.3011G>A, p.Ser1044Asn) (Figure 3A). We repeated the deep-sequencing of the sample using the other probe set to confirm the allele drop-out phenomenon as the source of error. The missed SNV was then identified with an allele coverage of 4,000 × and a variant frequency of 45%. We confirmed that the missed SNV existed in a cis-configuration adjacent to the rare SNV (Figure 3B).

### Precision performance of Ion Torrent S5 XL sequencing for SNV and small indel variant detection

To confirm the reproducibility of variant calling, the indel variant and SNV calling results from split samples are summarized in Table 3. Three indel variants and 43 SNVs were correctly called within and between runs; therefore the calling reproducibility was 100%. Precision performance was assessed by the coefficient of variation (CV) of the variant frequency using true heterozygote variants from three patients (Table 4). The results showed good precision performance, with the CV between 0.32 and 5.29% for indels, and between 3.0 and 3.86% for SNVs.

**Table 3:** Reproducibility of indel variants and single nucleotide variant (SNV) detection

Sample: variant	Replicates			Expected variants			Called variants			Reproducibility, %
	1st batch	2nd batch	3rd batch	1st batch	2nd batch	3rd batch	1st batch	2nd batch	3rd batch	
YMC1: *BRCA1*, c.5496_5506delinsA	1	1	3	1	1	3	1	1	3	100
YMC2: *BRCA2*, c.2798_2799del	1	1	3	1	1	3	1	1	3	100
YMC3: *BRCA1*, c.922_924delinsT	1	1	3	1	1	3	1	1	3	100
YMC1: 12 SNVs	1	1	3	12	12	36	12	12	36	100
YMC2: 13 SNVs	1	1	3	13	13	39	13	13	39	100
YMC3: 18 SNVs	1	1	3	18	18	54	18	18	54	100

SNV = single nucleotide variant; YMC = Yonsei Medical Center.

**Table 4:** Within-run and between-run precision performance for variant frequency calling

Sample: variant	Within-run				Total			
	Replicates	Mean, %	SD, %	CV, %	Replicates	Mean, %	SD, %	CV, %
YMC1: *BRCA1*, c.5496_5506delinsA	3	45.27	1.03	2.27	5	45.72	1.28	2.79
YMC2: *BRCA2*, c.2798_2799del	3	47.53	0.15	0.32	5	49.44	2.61	5.29
YMC3: *BRCA1*, c.922_924delinsT	3	49.53	1.01	2.04	5	50.40	2.08	4.13
YMC1: 8 heterozygote SNVs	3	49.89	1.56	3.12	5	49.64	1.86	3.76
YMC2: 7 heterozygote SNVs	3	49.36	1.69	3.42	5	49.69	1.92	3.86
YMC3: 14 heterozygote SNVs	3	50.09	1.50	3.00	5	50.06	1.79	3.57

YMC = Yonsei Medical Center; SD = standard deviation; CV = coefficient of variation.

## DISCUSSION

Here, we validated and optimized the Oncomine™ BRCA Research Assay and Ion Torrent S5 XL platform for routine *BRCA1/2* testing in a clinical laboratory. The previously noted shortcoming of the Ion Torrent platform is a higher error rate over the homopolymer region than in the Illumina platform [12]. Previous literature showed a significant increase in erroneous indel calls in homopolymer stretch of even 3–4 bp length [12]. Furthermore, a previous study with Miseq revealed homopolymer region-associated false positives in all tested samples with mean variant frequency of 21.6% [14]. However, in this validation study, we detected no homopolymer region-associated false positives at variant frequency cut-off of ≥ 20%. In ROI of this study, there was a total of 398 homopolymer regions (4–10 nucleotide length), which accounted for 10.1% of all target regions (1,797/177,21 bp, information on homopolymer stretches of ROI in this study are provided in the online version of Supplementary Table 2). A total of 66 true positive SNVs located in homopolymer region (47 heterozygote SNVs and 19 homozygote SNVs, 9.5% of total true positive variants) were all correctly called by our method. Our platform is considered suitable for analysis of genes with only relatively short homopolymer regions, such as *BRCA1/2*. Further validation is needed for homopolymer regions longer than 11 bp.

The implemented bioinformatics pipeline, Torrent Suite software with Torrent Variant Caller, was easy to use and showed optimal performance. Time and labor requirements for the entire process, including wet procedures and bioinformatics analysis, were reduced in our laboratory (comparison of wet-procedure time required for Sanger sequencing and next-generation sequencing is provided in the online version of Supplementary Figure 1). Furthermore, our platform concurrently detected CNVs, SNVs, and small indels. Since conventional Sanger sequencing cannot detect large deletion/duplication, a parallel copy number analysis testing, such as MLPA, is required. Our approach offers the benefit of reduced time and effort for CNV analysis.

A possible limitation of our study is that we only included small-sized indels, which were ≤ 11 bp. There were 7,965 reported variants in *BRCA1* and 9,816 variants in *BRCA2*, according to the dbSNP database (https://www.ncbi.nlm.nih.gov/SNP/). Among them, the number of variants with sizes that are not included in this study (11–400 bp) was 15 for *BRCA1* (0.41%) and 26 for *BRCA2* (0.15%), respectively. Although the proportion of large pathogenic indels in *BRCA1/2* is relatively low, further validation is required using indels that are larger than 11 bp.

A false-negative variant in this study was caused by the allele drop-out phenomenon from a rare variant at the primer-binding site. The allele drop-out phenomenon is known to be an error source in all polymerase chain reaction (PCR)-based techniques [15, 16]. If the rare and target variants exist in the trans-configuration, the target variant can be detected, even if one allele is dropped during PCR amplification. However, in our case, the rare and missed variants existed in the cis-configuration; the missed variant was detected only in other amplicons and accounted for only part of the total reads. When the variant frequency cut-off was decreased from 20% to 10%, a missing variant was called. However, false positive rate increased from 0.14% (one false-positive call) to 6.82% (51 false-positive calls). Therefore, we chose a variant frequency cutoff of 20%, and checked for missing true-positive calls in cases of samples with no pathogenic variant.

In conclusion, the Oncomine™ BRCA Research Assay used with the Ion Torrent S5 XL platform showed acceptable analytical sensitivity, specificity, accuracy, and precision performance for the detection of SNVs, small indels, and CNVs. The bioinformatics pipeline using Torrent Suite software with Torrent Variant Caller was optimized for the analysis of S5 XL data. In analysis of *BRCA1/2* genes, we confirmed that the error associated with homopolymer region is not a major problem in this new Ion Torrent assay. Further validation study is needed to evaluate the performance for longer homopolymer regions. It is important to note that a rare individual variant can be a source of error because it causes the allele drop-out phenomenon in deep sequencing, as with other PCR-based methods. NGS is a reliable and practical tool for clinical *BRCA1/2* testing.

## MATERIALS AND METHODS

### Patients and DNA samples

We included 40 Korean HBOC patients who had previously been subjected to Sanger sequencing and Illumina's MiSeq platform for *BRCA1/2* genetic variants [14]. Among the cohort samples, difficult cases with false negative or false positive calls from MiSeq were selected for this study. Ultimately, we included 12 samples with pathogenic variant, 11 samples with variants of unknown significance, and 17 samples with likely benign/benign variants. Since the 40 samples only had SNVs and small indels that were either smaller than or equal to 11 bp, we added three more samples with *BRCA1* pathogenic CNV that had been confirmed by multiplex ligation-dependent probe amplification (MLPA) to evaluate the performance of CNV detection. This study was approved by the hospital institutional review board, and informed consent was obtained from each patient. Genomic DNA was extracted from whole blood using a QIAamp DNA Blood Mini Kit (Qiagen, Venlo, The Netherlands). Each DNA sample was checked for purity using a NanoDrop 1000 system (Thermo Fisher Scientific) and for concentration using a Qubit 3.0 Fluorometer (Thermo Fisher Scientific). The concentration of input DNA was then adjusted to 0.67 ng/μL. The MLPA was performed using P002-D1 *BRCA1* and P045 *BRCA2/CHEK2* probemixes (MRC-Holland, Amsterdam, The Netherlands) according to the manufacturer's instructions. The MLPA result was analyzed using GeneMarker software (Softgenetics, State College, PA, USA).

### Library preparation and Ion S5 XL sequencing

Overall library preparation was carried out using an Ion Chef System (Thermo Fisher Scientific) according to the manufacturer's instructions. Briefly, barcoded libraries were generated from 10 ng of DNA per sample using an Ion AmpliSeq Chef Solutions DL8 Kit (Thermo Fisher Scientific) and an Oncomine™ BRCA Research Assay (Thermo Fisher Scientific). Two premixed pools of 265 primer pairs were used to generate the sequencing libraries. Clonal amplification of the libraries was carried out by emulsion PCR using an Ion AmpliSeq IC 200 Kit (Thermo Fisher Scientific). The prepared libraries were then sequenced on an Ion S5 XL Sequencer using an Ion 520 Chip and an Ion 520 kit–Chef Kit (all Thermo Fisher Scientific). Fifty-two tests were conducted in four separate batches by two different technologists working in shifts (only three samples with CNV were tested in another batch). Split samples from three patients with indel variants, which represent important and difficult case, were used to assess between-run and within-run precision performance. DNA samples were divided into aliquots and assayed (once in the first and second batches, and with three replicates in the third batch) to evaluate precision performance.

### Bioinformatics analysis

Generated raw sequence data in FASTQ format were aligned to the hg19 human reference genome using the Torrent Mapping Alignment Program aligner implemented in v5.2 of the Torrent Suite software (Thermo Fisher Scientific). For SNV calling, we used two software programs in parallel—plug-in Torrent Variant Caller v5.2.0.34 (Thermo Fisher Scientific) and commercial NextGENe v2.4.1.2 (Softgenetics)—to generate a variant call format file. For Torrent Variant Caller analysis, default setting of germline low-stringency parameters (minimal variant frequency of 0.1, minimum variant quality of 10, minimum coverage of 5 ×, maximum strand bias of 0.98, and minimum variant score of 10) was used and candidate variants were obtained only when variant frequency at a given position of ≥ 20% and variant coverage of ≥ 20×. For NextGENe analysis, candidate variant was called using the following setting parameters: minimum variant frequency of 0.1; minimum SNV balance ratio (number of forward reads/ number of reverse reads) of 0.1; and minimum homopolymer indel balance ratio of 0.8. Candidate variants were obtained after filtering variant of low coverage (< 20×). For equivocal variant calls from Torrent Variant Caller and NextGENe, Integrative Genomics Viewer v2.3 software (Broad Institute, Cambridge, MA, USA) and NextGENe Viewer (SoftGenetics) were used to visualize the sequence reads and alignments and identify sequencing errors, respectively.

CNV analysis was performed with dispersion and Hidden Markov Model (HMM) method with normalized counts in NextGENe software. For dispersion and HMM method, one sample file and another control file were required. We used a CNV-negative sample, confirmed by MLPA, as a control. CNV tool calculated the coverage ratios for each region, as well as the amount of dispersion (noise) for each region. We assigned a minimum dispersion value of 0.01 and a minimum region length of 60 in our default setting (minimum normalized read counts of 100 and estimated sample purity 100%). Using the coverage ratio value and the amount of noise in each region, copy number state of each region in the sample was reported (duplication/normal/deletion).

### Accuracy evaluation compared with Sanger sequencing and MLPA

All SNVs and small indels identified by NGS were compared with the Sanger sequencing results by setting the ROI to ± 20 bp of the exon/intron boundaries in each coding exon. The entire length of the ROI of *BRCA1* was 6,450 bp and that of *BRCA2* was 11,271 bp. The sensitivity of NGS was calculated as the number of true-positive NGS calls divided by the number of all sequence variants detected by Sanger sequencing. The specificity was calculated as the number of true-negative (wild-type) NGS calls divided by the number of wild-type bases by Sanger sequencing. The false-positive rate was calculated as the number of false-positive NGS calls divided by the number of false-positive plus the number of true-positive calls. The accuracy was calculated as the number of true-positive calls plus the number of true-negative calls divided by all NGS calls. Results from CNV analysis were compared with MLPA results.

## SUPPLEMENTARY MATERIALS FIGURES AND TABLES





## Abbreviations

NGS: next-generation sequencing
dPGM: Personal Genome Machine
indel: insertion/deletion
HBOC: hereditary breast and ovarian cancer syndrome
ROI: region of interest
SNV: single nucleotide variant
CNV: copy number variant
CV: coefficient of variation
PCR: polymerase chain reaction
MLPA: multiplex ligation-dependent probe amplification
HMM: Hidden Markov Model
GATK: genome analysis toolkit
CI: confidence interval
