# Supplementary material for: Validation and optimization of the Ion Torrent S5 XL sequencer and Oncomine workflow for BRCA1 and BRCA2 genetic testing
Source: Oncotarget. 2017 Apr 3;8(21):34858–66. doi: 10.18632/oncotarget.16799 (PMC5471017; doi:10.18632/oncotarget.16799)
Supplement: Supplementary file 1 [file oncotarget-08-34858-s001.pdf]

Validation and optimization of the Ion Torrent S5 XL sequencer and Oncomine workflow for *BRCA1* and *BRCA2* genetic testing

Supplementary Materials

Supplementary Table 1: Variant profile of the 43 samples used for NGS validation. See Supplementary_Table_1

Supplementary Table 2: Information of homopolymer stretches in region of interest in this study[a]

Homopolymer length (bp)	*BRCA1* No. of homopolymer region	*BRCA1* Total length of homopolymer region (bp)	*BRCA1* No. of tested variant in homopolymer region	*BRCA2* No. of homopolymer region	*BRCA2* Total length of homopolymer region (bp)	*BRCA2* No. of tested variant in homopolymer region
4	173	692	5 heterozygote SNVs	86	344	19 homozygote SNVs, 41 heterozgote SNVs
5	79	395	-	20	100	1 heterozygote SNVs
6	17	102	-	6	36	-
7	9	63	-	2	14	-
8	3	24	-	1	8	-
9	1	9	-	0	0	-
10	1	10	-	0	0	-
Total	283	1295	5 heterozygote SNVs	115	502	19 homozygote SNVs, 42 heterozgote SNVs

SNV, single nucleotide variant.
[a]Coding exons and ± 20 bp of flanking introns of *BRCA1* and *BRCA2*.

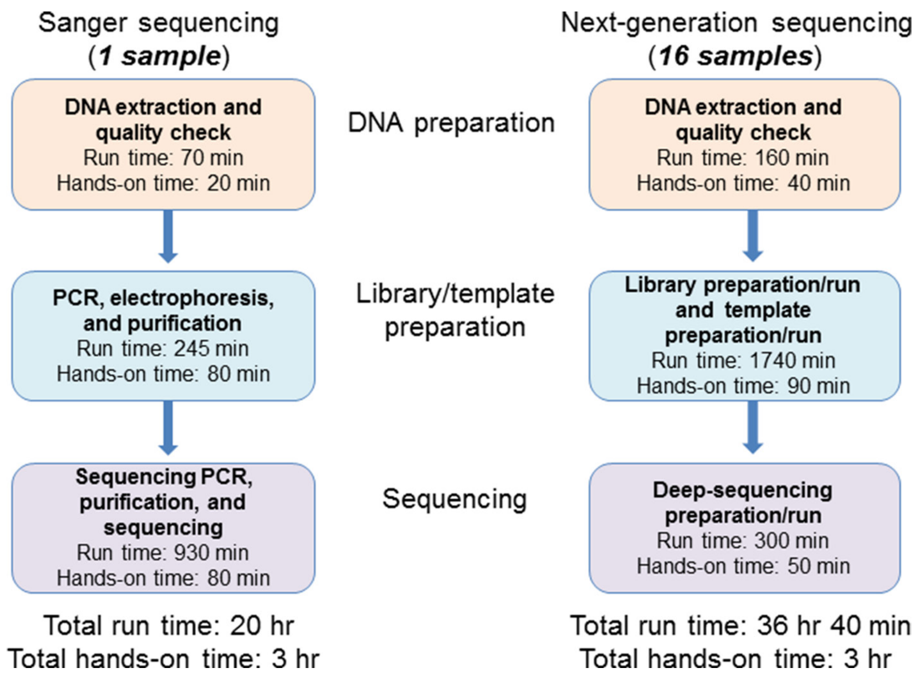

Supplementary Figure 1: Comparison of wet-procedure time required for Sanger sequencing and next-generation sequencing.
